# A Cell-Permeant Amiloride Derivative Induces Caspase-Independent, AIF-Mediated Programmed Necrotic Death of Breast Cancer Cells

**DOI:** 10.1371/journal.pone.0063038

**Published:** 2013-04-30

**Authors:** Leonardo J. Leon, Nagarekha Pasupuleti, Fredric Gorin, Kermit L. Carraway

**Affiliations:** 1 Department of Biochemistry and Molecular Medicine, UC Davis School of Medicine, Sacramento, California, United States of America; 2 Department of Neurology, UC Davis School of Medicine, Sacramento, California, United States of America; 3 UC Davis Comprehensive Cancer Center, UC Davis School of Medicine, Sacramento, California, United States of America; Roswell Park Cancer Institute, United States of America

## Abstract

Amiloride is a potassium-sparing diuretic that has been used as an anti-kaliuretic for the chronic management of hypertension and heart failure. Several studies have identified a potential anti-cancer role for amiloride, however the mechanisms underlying its anti-tumor effects remain to be fully delineated. Our group previously demonstrated that amiloride triggers caspase-independent cytotoxic cell death in human glioblastoma cell lines but not in primary astrocytes. To delineate the cellular mechanisms underlying amiloride’s anti-cancer cytotoxicity, cell permeant and cell impermeant derivatives of amiloride were synthesized that exhibit markedly different potencies in cancer cell death assays. Here we compare the cytotoxicities of 5-benzylglycinyl amiloride (UCD38B) and its free acid 5-glycinyl amiloride (UCD74A) toward human breast cancer cells. UCD74A exhibits poor cell permeability and has very little cytotoxic activity, while UCD38B is cell permeant and induces the caspase-independent death of proliferating and non-proliferating breast cancer cells. UCD38B treatment of human breast cancer cells promotes autophagy reflected in LC3 conversion, and induces the dramatic swelling of the endoplasmic reticulum, however these events do not appear to be the cause of cell death. Surprisingly, UCD38B but not UCD74A induces efficient AIF translocation from the mitochondria to the nucleus, and AIF function is necessary for the efficient induction of cancer cell death. Our observations indicate that UCD38B induces programmed necrosis through AIF translocation, and suggest that its cytosolic accessibility may facilitate drug action.

## Introduction

Most currently employed cancer therapeutics initiate apoptotic death in actively proliferating cancer cells. While such agents exhibit a significant degree of efficacy, two key barriers to more effective cancer treatment remain. First, because therapeutic regimens tend to target rapidly proliferating cells, any subset of cells that are dormant or that proliferate slowly can evade therapeutic intervention and give rise to either primary tumor recurrence or the emergence of metastatic lesions [1]. Second, tumor cells commonly activate potent anti-apoptotic pathways to promote their survival and drug resistance [2]. Thus, the development of small molecules that act independently of cell cycle progression to engage non-apoptotic cell death mechanisms offers a particularly attractive approach to thwart tumor progression [3].

Numerous studies in recent years point to the existence of programmed cell death (PCD) mechanisms that are distinct from caspase-dependent (type I) apoptosis [4]–[7]. For example, autophagy, or type II PCD, is a stress-induced salvage pathway employed by cells experiencing limited nutrients. Through this mechanism cells sequester bulk cytoplasm and organelles into double membrane vesicles, which ultimately fuse with lysosomes to mediate the degradation of their contents and provide nutrients to support cell survival [8], [9]. However, if the stressful conditions become overwhelming the type II PCD pathway can trigger caspase-independent cell death.

Historically, necrosis has been conceptualized as a non-specific cell death process, involving the disruption of the plasma membrane and extrusion of the cytosolic contents, with the potential induction of inflammatory response. However, very recent studies indicate that some necrotic processes, such as programmed necrosis (type III PCD), are under the control of the cell and contribute to both physiological and pathological processes [10], [11]. While many of the molecular and cellular details of programmed necrosis remain to be elucidated, it is now recognized that programmed necrosis can be orchestrated by key cellular factors such as the mitochondrial flavoprotein apoptosis-inducing factor (AIF), and is characterized by the swelling of organelles such as mitochondria and the endoplasmic reticulum followed by the loss of plasma membrane integrity.

Amiloride, an FDA-approved diuretic that acts on epithelial sodium channels, has been demonstrated in numerous studies to suppress the growth and metastasis of a variety of tumor types in rat and mouse models (reviewed in [12]). We have demonstrated that high-dose amiloride treatment is cytotoxic toward cultured glioma cell lines but does not affect primary rat astrocytes at the same concentration, and that cytotoxicity is caspase-independent and independent of amiloride’s inhibitory activities toward the type 1 sodium-proton exchanger (NHE1) and the sodium-calcium exchanger (NCX) [13], [14]. Moreover, the amiloride derivatives 5-(N-ethyl-N-isopropyl)-amiloride (EIPA) and hexamethylene amiloride (HMA) have been reported to reduce the growth, viability, motility and invasiveness of hepatocellular carcinoma cells and xenografts [15]–[17]. HMA has also been shown to induce cell death in leukemic cells, while not affecting the viability of normal hematopoietic cells [18]. Taken together, these observations suggest that amiloride and its derivatives exhibit selective anti-cancer cytotoxicity independent of its ion channel inhibitory activity, making this class of drugs attractive for future clinical evaluation.

A major challenge to repurposing amiloride as an anti-cancer therapeutic is its low potency in cytotoxicity assays. In the present study we have examined the breast cancer cell cytotoxicity of two amiloride derivatives modified at the C(5) position with different substituents. Surprisingly, we observed that the more potent of these derivatives induces cell death via AIF-mediated programmed necrosis, raising the possibility that such amiloride derivatives may be employed to attack tumor cells inherently resistant to apoptosis-inducing therapeutics. Moreover, its cytotoxic activity appears to correlate with its cell permeability, suggesting the existence of a novel intracellular target for amiloride and its derivatives.

## Materials and Methods

### Reagents and Chemicals

UCD74A and UCD38B were synthesized, purified and handled as previously described [19]. Amiloride (Sigma, St. Louis, MO), UCD74A, and UCD38B were dissolved in DMSO as 200 mM stocks, cisplatin (Calbiochem) as a 20 mM stock, and ionomycin (Calbiochem) as a 5 mM stock. Compounds were stored in the dark at −20°C. The pancaspase inhibitor z-VAD-fmk (Biovision, Mountain View, CA) was used at 1 µM concentration and treatments were carried out for 24 hours. The Green Multi-Caspase Staining kit was purchased from PromoKine and was employed according to the manufacturer’s instructions. The autophagy inhibitors chloroquine and compound C (Sigma) were used at 10 µM and 25 µM, respectively, and treatment was carried out for 24 hours.

### Cell Culture

All cell lines were obtained from American Type Culture Collection. MDA-MB-231 and MCF-7 cells were cultured in Dulbecco’s modified Eagle’s medium, while SKBR3 cells were cultured in McCoy’s 5A medium. Media contained 10% bovine fetal calf serum and 2% v/v penicillin/streptomycin. Cells were maintained at 37°C in a 10% CO_2_ humidified incubator.

### Immunoblot Analysis

After treatment with inhibitors, cells were lysed in SDS sample buffer, lysates were resolved by 12% SDS-polyacrylamide gel electrophoresis, and proteins transferred to nitrocellulose. Membranes were blocked for 1 hour with 5% milk in Tris-buffered saline/0.05% Tween-20 (TBST) followed by overnight 4°C incubation in primary antibodies. Blots were then washed with TBST and incubated with HRP-conjugated secondary antibody (goat anti-mouse or anti-rabbit IgG, Invitrogen-Molecular Probes) for one hour at room temperature. Visualization of chemiluminescence signal was carried out using an Alpha Innotech Digital Imaging Station. Primary antibodies employed include PARP-1, cleaved caspase-7, LC3, GAPDH, CREB (Cell Signaling Technology, MA), actin (Sigma, St. Louis, MO), CyclinD1 (Thermo Scientific, Fremont, CA) and Beclin1 H-300 and AIF B-9 (Santa Cruz Biotechnology, CA).

### Live Cell (MTT) Tetrazolium Assay

Cells were seeded in 24-well plates and grown overnight, and then treated with various compounds for an additional 24 hours. Cells were then treated with 0.5 mg/ml 3-(4,5-Dimethyl-thiazol-2-yl)-2,5-diphenyltetrazolium bromide (MTT) for 2 hours at 37°C, media was removed, and formazan crystals were dissolved with 300 µl of acidic isopropyl alcoholic solution. 200 µl of sample was transferred to a 96-well plate, and absorbance was measured at 570 nm with 655 nm reference. The absolute optical density was normalized to time-matched, vehicle-treated control cells and expressed as percent viability.

### Cytotoxic Lactate Dehydrogenase (LDH) Assay

Cells were seeded in 24-well plates and grown overnight. After treatment with and without inhibitors, 100 µl of supernatant per well was transferred into a 96-well plate and cellular LDH release from injured cells was determined using the LDH Cytotoxicity Detection Kit (Clontech) according to the manufacture’s instructions. Treated cells were normalized to time-matched positive control cells treated with 1% Triton X-100 (Sigma, MO).

### Fluorescence Microscopy

The three amiloride derivatives exhibit similar intrinsic fluorescence (unpublished observations), and their visualization was carried out as previously described [20] using an Olympus BX61 deconvolving fluorescence microscope with excitation at 350 nm and emission at 460 nm. For AIF localization studies, cells were cultured in chamber slides overnight, and then treated with DMSO or 250 µM UCD38B, UCD74A or amiloride for 2 hours at 37°C. Cells were washed with PBS and fixed with 4% paraformaldehyde at room temperature for 15 minutes, then washed with PBS and permeabilized with 80% methanol in PBS for 5 minutes. Cells were blocked with 3% non-fat dried milk/1% BSA in PBS for 1 hour at room temperature and incubated with 1∶100 anti-AIF mAb in 0.1% BSA/PBS overnight at 4°C. Alexa Fluor 488-conjugated anti-mouse IgG (Invitrogen-Molecular Probes, Eugene, OR) was used at 1∶500 dilution in 0.1% BSA/PBS for 1 hour at room temperature. After PBS wash, slides were mounted with mounting media containing 4′,6-diamidino-2-phenylindole (DAPI; Invitrogen-Molecular Probes). Imaging was carried out using a confocal microscope (Olympus).

### Electron Microscopy

Cells were plated in 8-well chambered slides overnight and then treated with 250 µM UCD38B, UCD74A or amiloride for 2 hours at 37°C. Cells were fixed in Karnovsky fixative and were imaged using a Philips 120 BioTwin electron microscope at 80 KV equipped with a Gatan MegaScan model 794/20 digital camera.

### Cellular Fractionation

After treatment with inhibitors, cells were trypsinized, spun at 300×g for 5 minutes, resuspended in hypotonic buffer (20 mM Tris-HCl pH 7.4, 10 mM NaCl, 3 mM MgCl_2_), transferred to a pre-chilled microcentrifuge tube and incubated on ice for 15 minutes. 25 µl 10% NP-40 was added and the tube was vortexed for 10 seconds. The homogenate was centrifuged at 1800×g at 4°C and both the supernatant (cytosolic fraction) and pellet (nuclear fraction) were collected. The nuclear fraction was resuspended in 50 µl extraction buffer (100 mM Tris/HCl, pH 7.4, 2 mM Na_3_V0_4_, 100 mM NaCl, 1% Triton X-100, 1 mM EDTA, 10% glycerol, 1 mM EGTA, 0.1% SDS, 1 mM NaF, 0.5% deoxycholate, 20 mM Na_4_P_2_O_7_, 1 mM AEBSF, 4 µg/ml pepstatin A, 4 µg/ml leupeptin, 4 µg/ml aprotinin) for 30 minutes on ice with vortexing for 5 second every 10 minutes. The sample was then centrifuged for 30 minutes at 14,000×g at 4°C and the supernatant (nuclear extract) was collected. Cytosolic and nuclear samples were treated with SDS sample buffer and immunoblotting analysis was performed as described above.

### siRNA Knockdown

Cells were seeded in 24-well plates and were grown for 16 hours. Cells were then transfected with 100 nM ON-TARGETplus SMARTpool RNAi oligonucleotides (Dharmacon) directed to AIF, cyclinD1, or beclin1, or with nontargeting siRNA per manufacturer’s instructions. Proliferation assays and immunoblotting were carried out after 48 hours as described above.

### FACS Assessment of Necrosis

MDA-MB-231 cells were treated with DMSO, 250 µM UCD38B, or 50 µM cisplatin (CP) for 24 hours, and suspended cells were incubated with FITC-Annexin V and propidium iodide (PI) from BD Pharmingen FITC Annexin V Apoptosis Detection Kit 1 as described by the manufacturer. Cells were sorted using a Becton Dickinson FACScan flow cytometer, data were collected using Cell Quest version 3.3 software and analyzed using FloJo version 7.6.5 software.

### Statistical Analysis

Values are expressed as mean ± standard error and were calculated from experiments carried out in triplicate. *P* values were determined using the independent two sample t-test with values less than 0.05 considered statistically significant (*, *P*<0.05; **, *P*<0.001).

## Results

### Cytotoxicity of Amiloride Derivatives Correlates with Cell Permeability

**Figure 1:**
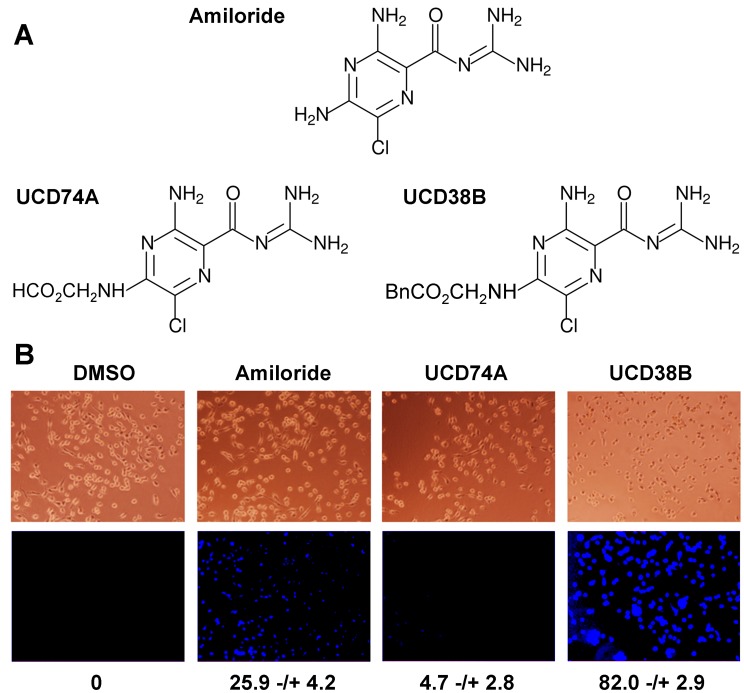
Differential cell permeability of amiloride derivatives. (**A**) The structures of amiloride and the two C(5) derivatives employed in these studies are depicted. The Bn in the UCD38B structure refers to a benzyl group. (**B**) MDA-MB-231 cells were incubated with vehicle control or 250 µM amiloride, UCD74A or UCD38B for 10 minutes and rinsed with PBS. Treated cells were then imaged by brightfield (upper panels) or fluorescence (lower panels) microscopy, the fluorescence intensities of 20 randomly selected cells were quantified, and averages with standard error are indicated.

To begin to develop amiloride derivatives that exhibit greater potency in tumor cell death assays, we have replaced the C(5)-amino group with glycine to make compound UCD74A, or additionally added benzyl alcohol to the glycine carboxylate to make the more hydrophobic compound UCD38B [20] (see Figure 1A). Surprisingly, we observed that the cytotoxic potencies of these compounds correlate with their cell permeabilities. Using the intrinsic fluorescence of all three compounds to assess their cellular uptake by fluorescence microscopy (Figure 1B), we observed that amiloride modestly accumulates within MDA-MB-231 breast tumor cells, UCD38B is far more permeant, while UCD74A appears to be largely excluded from the cell interior. These observations indicate that modification of the 5-amino group of amiloride can markedly alter the cell permeability of analogs.

**Figure 2:**
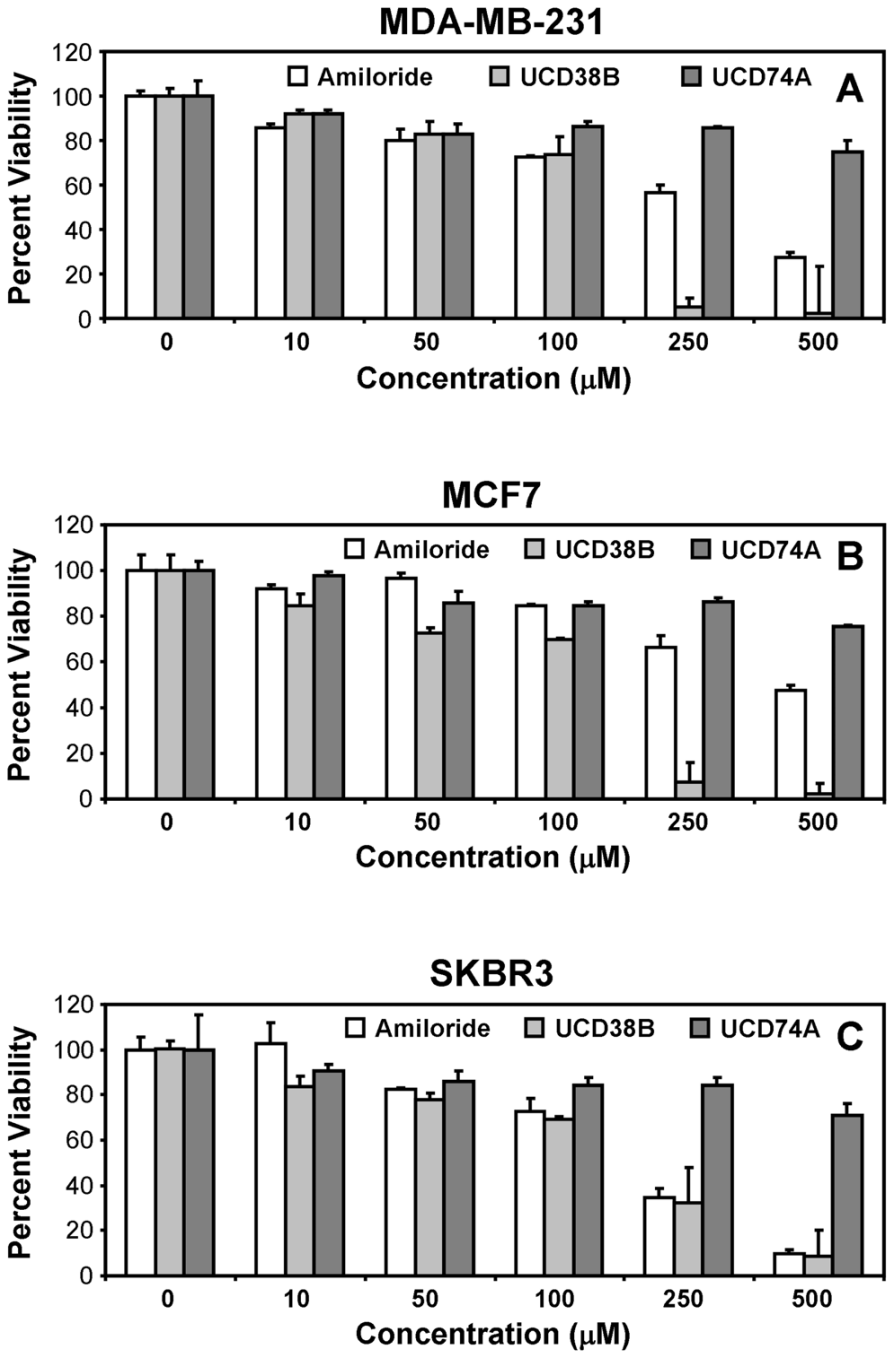
Induction of breast tumor cell death by the amiloride derivative UCD38B. (**A**) MDA-MB-231, (**B**) MCF7, and (**C**) SKBR3 breast cancer cells were exposed for 24 hours to various concentrations of amiloride, UCD74A, or UCD38B, as indicated. Cell viability was measured using the MTT assay. Results depicted in each panel are representative of three independent experiments.

To assess the impact of the derivatives on tumor cell viability, we first examined a panel of cultured human breast cancer cell lines using the MTT assay. Breast cancer is a general descriptor applied to several genetically and pathologically distinct diseases that may be distinguished using the biomarkers HER2, estrogen receptor (ER), and progesterone receptor (PR), and strategies for treatment can vary markedly depending on classification. SKBR3 cells are a model of HER2-positive breast cancer, MCF7 cells are a model of ER/PR-positive breast cancer, and MDA-MB-231 cells are a model of triple-negative or basal breast cancer. While we observed some variability among the model cell lines, each line was sensitive to amiloride (Figure 2) at concentrations previously observed to kill cultured human glioma cells [13], [14], suggesting that the drug may act broadly toward multiple tumor types and subtypes. We observed that amiloride exhibits a modest potency toward each of the cell lines, eliciting half-maximal effects at 100–200 µM and full effects at >500 µM. UCD38B is more than twice as potent as amiloride in all cell lines, while UCD74A is relatively inert (Figure 2). These observations indicate that the cytotoxic potency of amiloride can be modulated by modification of its C(5) position. Moreover, the results illustrated in Figures 1B and 2 strongly suggest that cell permeability may play a significant role in the cytotoxicity of the drug.

### UCD38B-induced Cell Death Occurs Independently of the Cell Cycle

**Figure 3:**
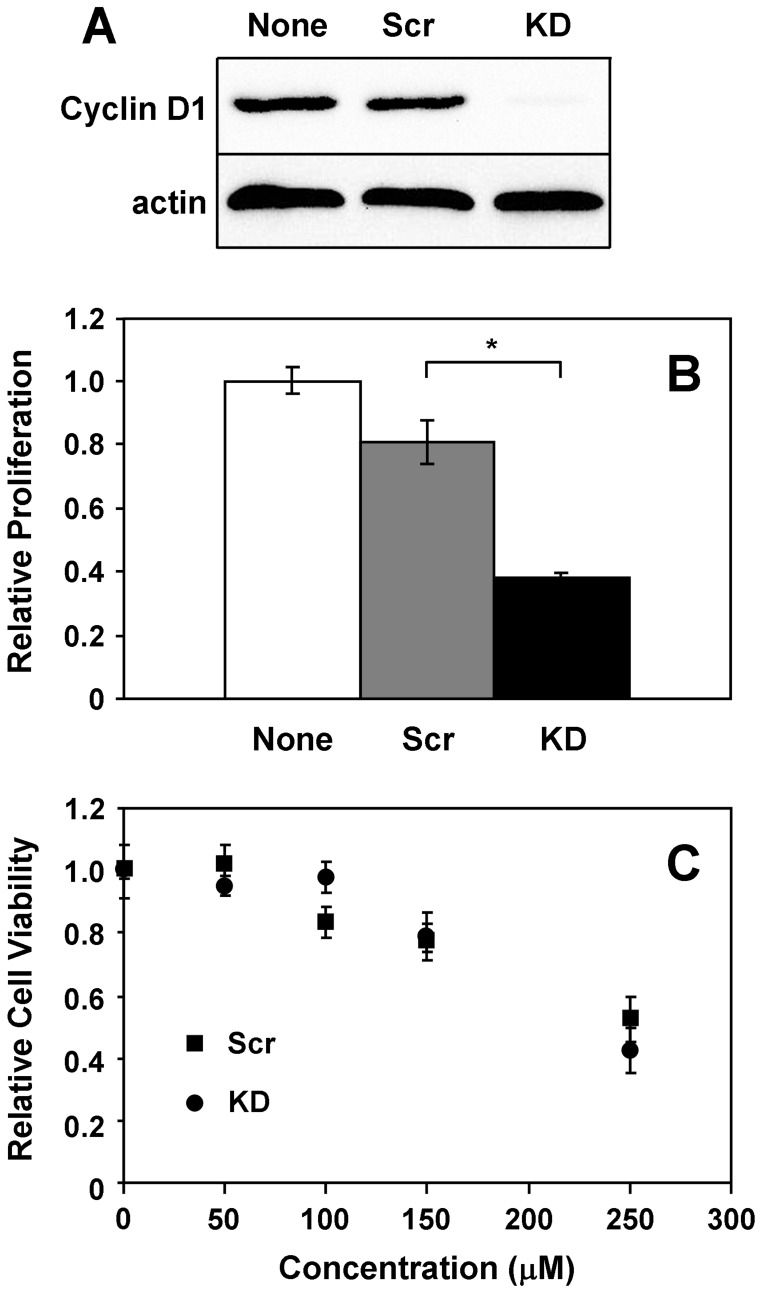
UCD38B-induced cell death occurs independently of the cell cycle and proliferation. MDA-MB-231 cells were left untreated (None) or were treated with scrambled (Scr) or cyclin D1 knockdown (KD) siRNA oligonucleotides for 72 hours. (**A**) Lysates were immunoblotted for cyclin D1 and actin proteins. (**B**) Cell proliferation after 24 hours was measured by MTT assay. (**C**) Cells treated with either scrambled control (squares) or knockdown (circles) oligonucleotides were exposed to increasing concentrations of UCD38B for 24 hours, and the fraction cell viability was determined using the MTT assay. Results depicted in each panel are representative of at least three independent experiments. *, *P*<0.05.

The majority of anti-cancer chemotherapeutics target cycling cells, limiting their efficacy toward dormant cells that could re-initiate primary breast tumors or seed metastatic lesions. To determine whether UCD38B cytotoxic effects are cell cycle-dependent, we examined the impact of cyclin D1 knockdown on UCD38B-induced cytotoxicity. Since amiloride and its derivatives exhibited qualitatively similar effects on all three breast cancer cell lines, subsequent cell death studies were limited to the highly malignant MDA-MB-231 line. siRNA-mediated cyclin D1 knockdown suppresses cyclin D1 protein levels in MDA-MB-231 cells by greater than 95% (Figure 3A), decreases the fraction of cells in S-phase from 14% to 5% (not shown), and suppresses proliferation as measured by MTT assay by greater than 50% (Figure 3B). However, impeding cell cycle progression at G1/S has no discernable effect on the ability of UCD38B to induce cell death (Figure 3C), indicating that proliferation is not a requirement for the cytotoxicity of this class of drugs.

### Apoptosis and Autophagy do not Account for UCD38B Cytotoxicity

**Figure 4:**
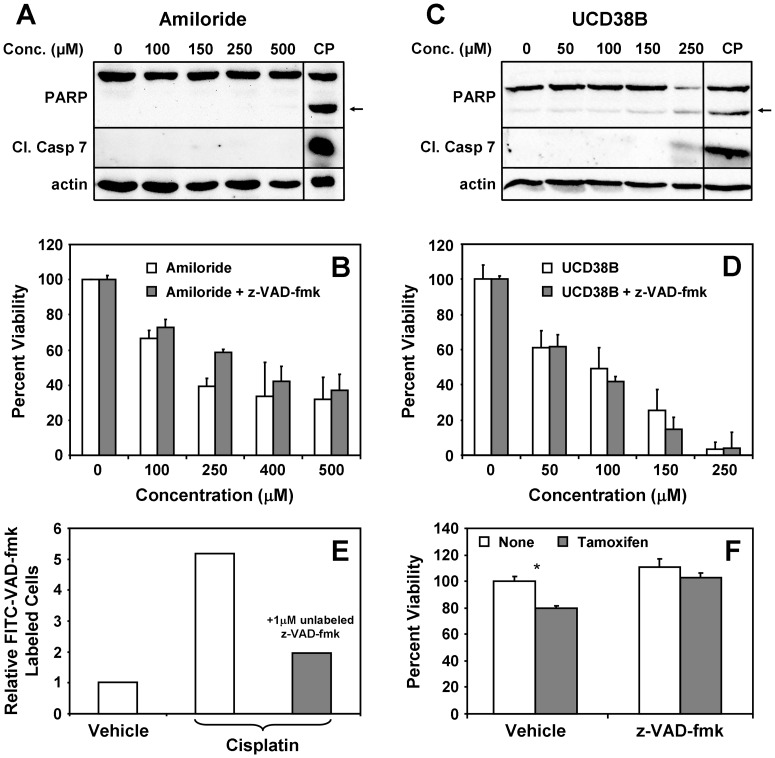
Amiloride and UCD38B do not induce breast tumor cell death through caspase-dependent apoptosis. MDA-MB-231 cells were treated for 24 hours with various concentrations of (**A,B**) amiloride or (**C,D**) UCD38B. (**A,C**) Cell lysates were immunoblotted with antibodies to PARP to assess cleaved PARP (arrow) formation, and with antibodies specific for cleaved caspase-7. CP indicates treatment with 50 µM cisplatin. (**B,D**) The viability of MDA-MB-231 cells after 24 hours was measured by MTT after treatment with various concentrations of amiloride or UCD38B in the presence and absence of 1 µM z-VAD-fmk. (**E**) Cells were treated without and with 50 µM CP for 24 hours in the presence of either DMSO control or 1 µM unlabled z-VAD-fmk to block caspase labeling. Cells were then treated with FITC-z-VAD-fmk, and stained cells determined and quantified by fluorescence microscopy. (**F**) Cells were treated with 5 µM tamoxifen in the presence and absence of 1 µM z-VAD-fmk, as indicated, and cell viability was measured using an MTT assay. Results depicted in each panel are representative of at least three independent experiments.

**Figure 5:**
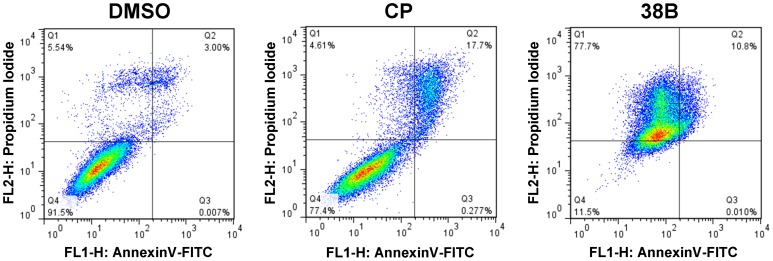
UCD38 does not significantly increase Annexin V staining. MDA-MB-231 cells were treated as indicated with DMSO control, 50 µM cisplatin (CP) as an inducer of caspase-mediated apoptosis, or 250 µM UCD38B, and then analyzed by FACS for annexin V binding and propidium iodide uptake. The accumulation of cells in quadrant (Q2) is characteristic of apoptosis while the accumulation of cells in quadrant 1 (Q1) is characteristic of necrosis.

To elucidate the mechanism of UCD38B-induced cell death, we first asked whether amiloride or UCD38B induce canonical caspase-dependent apoptosis. Consistent with our previous observations that amiloride promotes the caspase-independent death of cultured human glioblastoma cells [13], [14], we observed that amiloride does not induce PARP and caspase-7 cleavage in MDA-MB-231 breast tumor cells, and that co-incubation with the caspase inhibitor z-VAD-fmk does not significantly attenuate the cytotoxicity of amiloride (Figures 4A and 4B). Likewise, UCD38B induces only modest PARP and caspase-7 cleavage compared to the apoptosis-inducing agent cisplatin, and its cytotoxic potency is unaffected by z-VAD-fmk (Figure 4C and 4D). Under these conditions, z-VAD-fmk efficiently binds to caspases within cells (Figure 4E) and inhibits tamoxifen-induced MDA-MB-231 cells (Figure 4F) as previously described [21]. In addition, treatment of cells with UCD38B does not significantly promote staining of cells with FITC-Annexin V, a marker of apoptosis, but efficiently induces the uptake of propidium iodide, a marker of cell permeability and death (Figure 5). Taken together, these observations indicate that even though UCD38B very modestly induces PARP and caspase-7 cleavage, it acts through a cell death pathway that is largely independent of canonical apoptosis.

**Figure 6:**
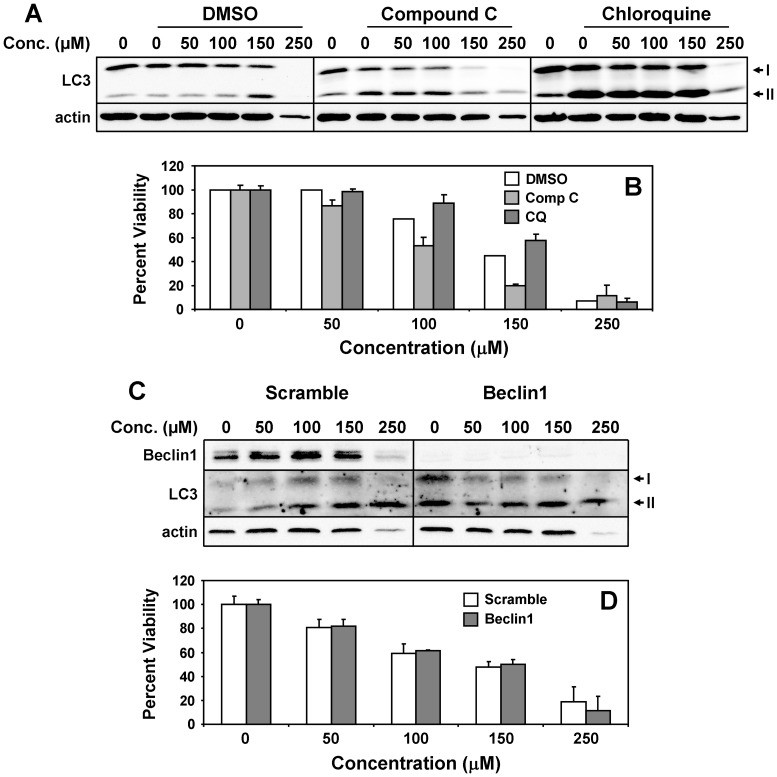
UCD38B does not cause cell death via autophagy. (**A,B**) MDA-MB-231 cells were treated for 24 hours with increasing concentrations of UCD38B in the presence of DMSO control, 10 µM Compound C, or 25 µM chloroquine (CQ). (**A**) Lysates were immunoblotted for LC3 and actin, and (**B**) viability was determined using the MTT cell death assay. (**C,D**) Cells were transfected with scrambled control or Beclin1-directed siRNA oligonucleotides, and (**C**) lysates were immunoblotted for beclin1, LC3 and actin, and (**D**) viability was determined using the MTT cell death assay.

Autophagy is a homeostatic cellular process that attempts to preserve cell viability under environmental stress, but can initiate cell death if conditions become too harsh [22]. UCD38B induces an autophagic response in MDA-MB-231 cells, as reflected in the elevation of the autophagy protein LC3 and its lipid modification to the LC3-II form (Figure 6A). However, inhibition of the autophagic process by the AMPK inhibitor compound C or the lysosomotropic agent chloroquine [23], [24] (Figures 6A and 6B), or by a greater than 90% knockdown of the autophagy protein beclin-1 (Figures 6C and 6D), does not disrupt UCD38B-induced cell death. Taken together, these observations indicate that autophagic cell death likely does not account for UCD38B anti-cancer cytotoxicity.

### UCD38B does not Induce Necrotic Cell Death through Calcium-mediated Calpain Activation

**Figure 7:**
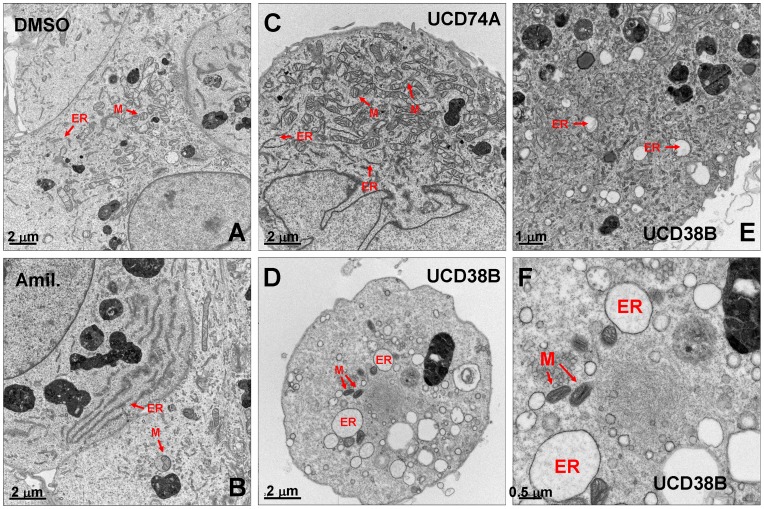
UCD38B treatment results in rapid swelling of the endoplasmic reticulum. MDA-MB-231 cells were treated with (**A**) DMSO control (x11357), (**B**) amiloride (x15455), (**C**) UCD74A (x15455) or (**D–F**) UCD38B (x15455, x20500, x38518) for two hours, and fixed cells were imaged by transmission electron microscopy. Examples of endoplasmic reticulum (ER) and mitochondrial (M) structures are indicated.

We have observed that UCD38B but not UCD74A triggers the formation of large intracellular vacuoles, membranous structures derived from intracellular organelles that are frequently formed in response to cell stress [6]. To determine whether UCD38B-induced cytotoxicity might involve structural changes to specific intracellular organelles, we carried out transmission electron micrographic analysis of MDA-MB-231 cells treated with DMSO control or with UCD74A or UCD38B for two hours. We observed that UCD38B (Figures 7D–F) but not UCD74A (Figure 7C) induces the massive dilation of the endoplasmic reticulum (ER) by one to two orders of magnitude, resulting in the formation of large cytosolic vacuoles in essentially every treated cell. Amiloride treatment induces similar changes as UCD38B (Figure 7B), except that morphological differences are far less pronounced. These observations indicate that the disruption of ER integrity is associated with UCD38B cytotoxicity.

**Figure 8:**
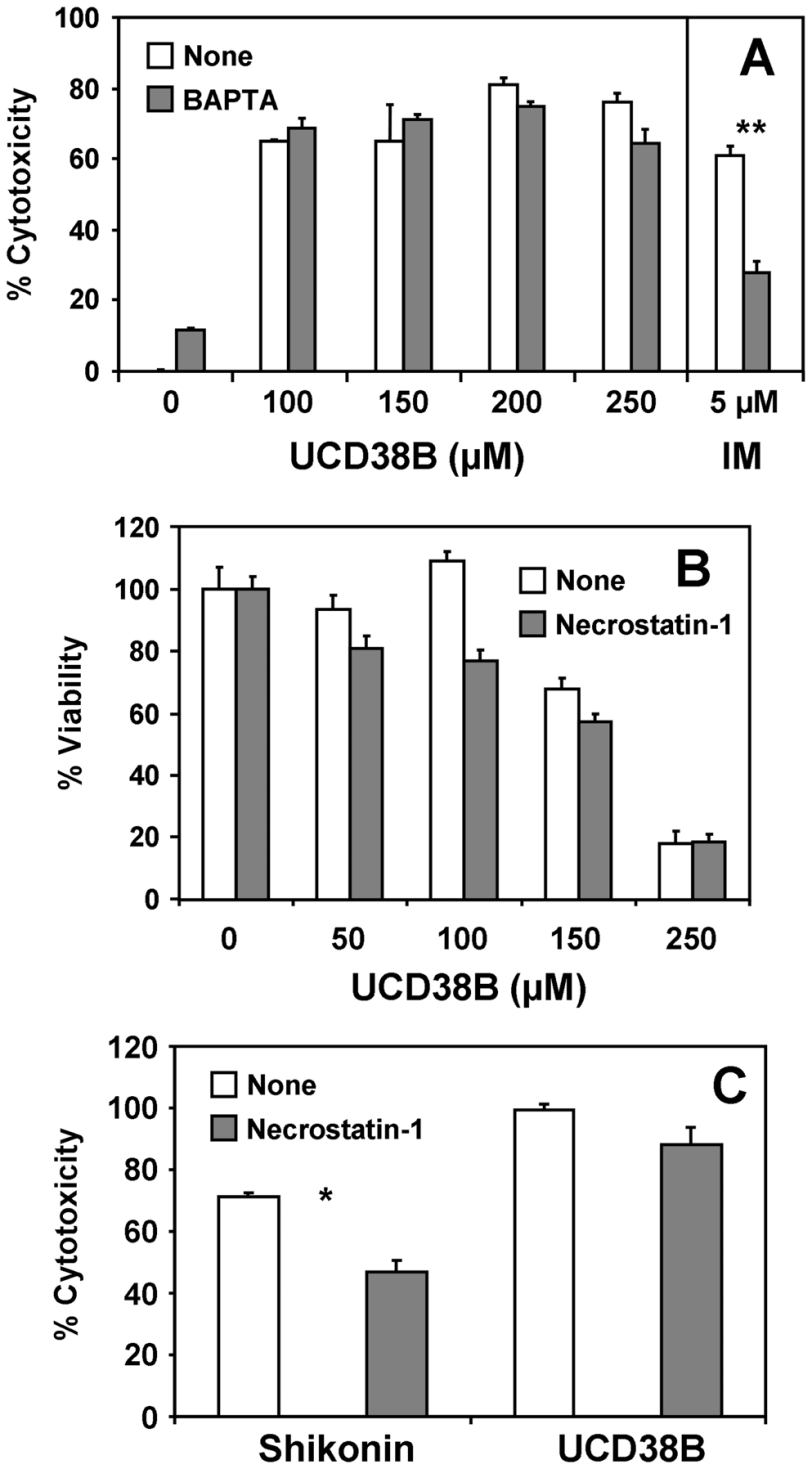
UCD38B does not induce calcium-dependent necrosis. (**A**) LDH cell death assays were carried out with MDA-MB-231 cells that were treated with UCD38B and ionomycin (IM) for 24 hours with and without 1 hour pre-treatment with 10 µM BAPTA-AM. (**B**) Cell viability after 24 hours was measured in the absence and presence of 25 µM RIP1 necroptosis inhibitor necrostatin-1 using the MTT assay. (**C**) LDH cell death assays were carried out with cells treated with 8 µM shikonin or 250 µM UCD38B in the presence and absence of 25 µM necrostatin-1. Results depicted in each panel are representative of three independent experiments. *, *P<0.05*; **, *P*<0.001.

We have recently demonstrated that cytosolic free calcium levels are elevated in glioblastoma cells relative to primary astrocytes, and that further calcium elevation induces cell death [14]. These observations raise the possibility that the observed structural alterations in the ER could trigger an increase in cytoplasmic free calcium, leading to calpain activation the induction of necrosis. This mechanism has been implicated in the dehydroeburicoic acid-induced necrotic death of glioblastoma cells [25]. To determine whether calcium-dependent necrosis contributes to UCD38B-dependent cytotoxicity, we examined cell death using the lactate dehydrogenase (LDH) release assay, a measurement of the extrusion of cytosolic contents from necrotic cells. We observed that UCD38B does indeed provoke the release of LDH from dying cells (Figure 8A), indicating that the compound likely induces a necrotic mechanism of cell death. However, pre-treatment of cells with the calcium chelator BAPTA-AM did not affect UCD38B-induced cell death under conditions where it inhibited ionomycin-induced cell death (Figure 8A). Moreover, treatment of cells with RIP1 inhibitor necrostatin-1 [26] did not affect cell death (Figures 8B) under conditions where it inhibits necroptic cell death by shikonin ([27] Figure 8C). Together, these observations strongly suggest that UCD38B induces a form of necrosis distinct from that mediated by calcium-induced calpain and RIP1-mediated necroptosis.

### UCD38B Induces AIF Nuclear Translocation

**Figure 9:**
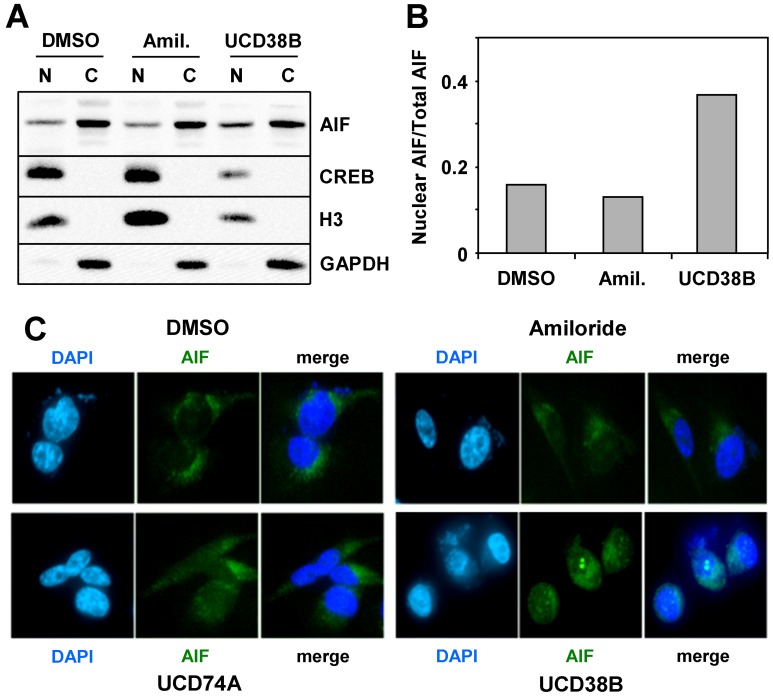
AIF translocates to the nucleus upon treatment of cells with UCD38B. (**A**) MDA-MB-231 cells were treated with 500 µM amiloride, or 250 µM UCD38B for 2 hours, and cells were fractionated into nuclear (N) and cytosolic (C) components. Fractions were immunoblotted for CREB and histone H3 as nuclear markers, GAPDH as a cytosolic marker, and AIF. (**B**) Bands from the blot depicted in (A) were digitally quantified, and the nuclear AIF as a fraction of the total AIF was plotted for each condition. (**C**) Cells were treated for 2 hours with DMSO control or 250 µM amiloride, UCD74A or UCD38B. Cells were then fixed and stained with DAPI to visualize the nucleus (blue) and antibodies to AIF (green), and examined by confocal fluorescence microscopy. Results depicted in each panel are representative of 2–3 independent experiments.

Since it has been previously suggested that AIF translocation from mitochondria to the nucleus initiates caspase-independent necrosis [28], we examined the localization of AIF without and with amiloride, UCD74A or UCD38B treatment by cellular fractionation (Figures 9A and 9B), and by confocal immunofluorescence microscopy (Figure 9C). Both assays revealed a significant translocation of AIF from the cytosol, presumably the mitochondria, to the nucleus specifically upon treatment with cytotoxic concentrations of 38B.

**Figure 10:**
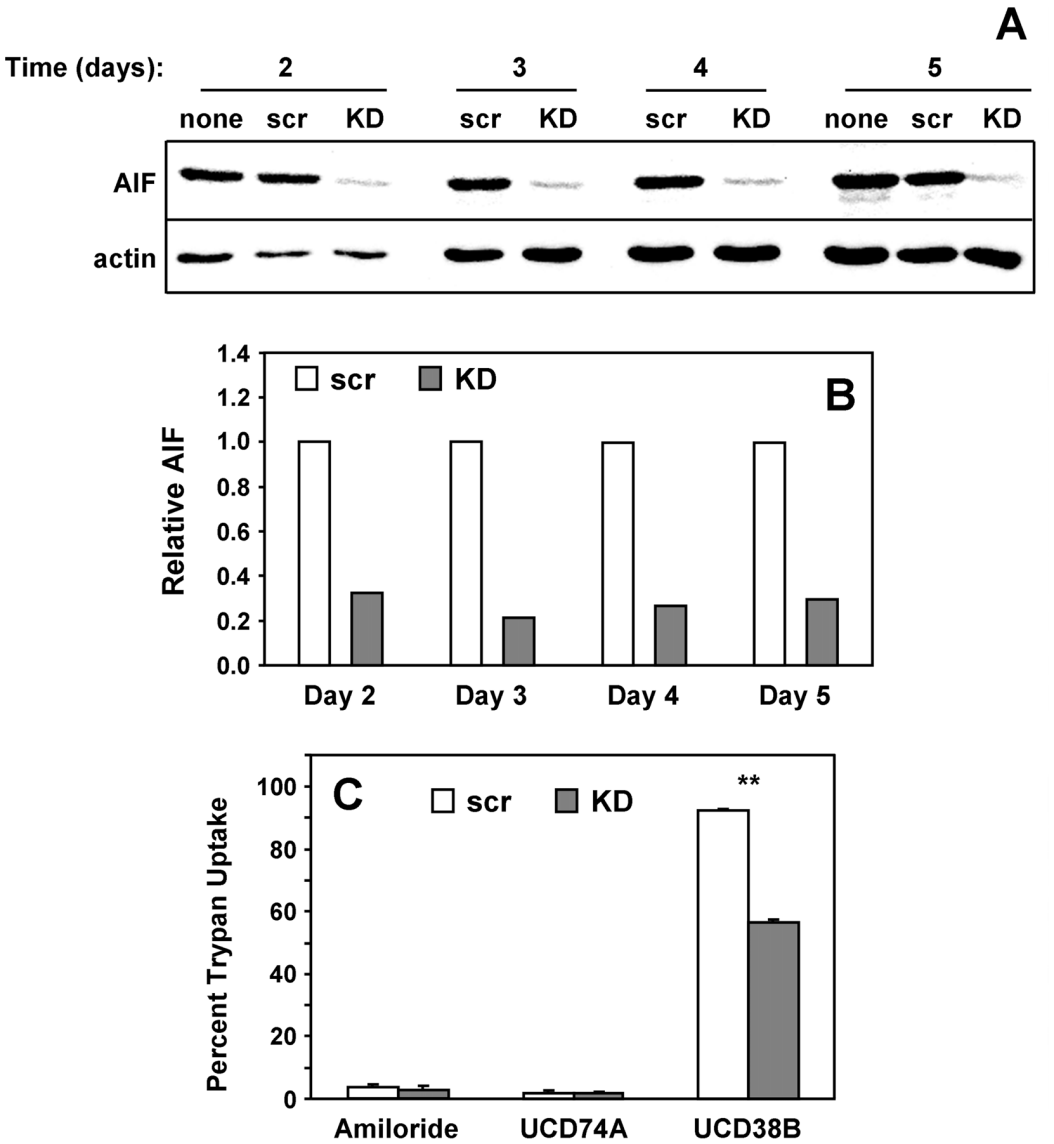
AIF is required for efficient UCD38B-induced cell death. (**A**) MDA-MB-231 cells were left untreated, or were treated with scrambled control (scr) or AIF-directed (KD) siRNA oligonucleotides for the indicated number of days. Cell lysates were immunoblotted for actin and AIF. (**B**) The blots from (A) were quantified and the relative amount of AIF (AIF/actin ratio) plotted. (**C**) Cells were treated with control or knockdown oligonucleotides for 3 days, then treated without or with 500 µM amiloride, 250 µM UCD74A or 250 µM UCD38B for another 24 hours, and the percent of cells that exhibit trypan blue uptake was evaluated. Results depicted in each panel are representative of three independent experiments. **, *P*<0.001.

To determine whether AIF contributes to cytotoxicity, we examined the impact of AIF knockdown on UCD38B-induced cell death. siRNA-mediated AIF knockdown led to 65–75% suppression of the protein over a course of 2–5 days (Figures 10A and 10B), and led to a reproducible reversal of UCD38B cytotoxicity toward MDA-MB-231 cells (Figure 10C). While the incomplete reversal of UCD38B-induced cell death is likely the result of incomplete AIF knockdown, these observations indicate that AIF contributes substantially to UCD38B cytotoxicity.

## Discussion

The current study builds upon our previous observations that amiloride selectively induces the caspase-independent death of glioma cells relative to non-transformed glial cells [13], [14]. Here we extend our analysis to examine the impact of two amiloride derivatives on breast cancer cell lines, allowing us to draw several key conclusions concerning the suitability of amiloride derivatives in attacking malignant tumors.

Our comparison of amiloride-based congeners UCD38B and UCD74A demonstrate that the modification of the C(5) amino group of the amiloride parent compound can significantly impact its cytotoxic activity; modification with the 5-benzylglycinyl moiety potentiates cytotoxicity while conversion of the C(5) amino group to glycine inhibits this function. These observations suggest that bulkier or more hydrophobic substituents at this position may augment cytotoxicity. Our future studies will be directed toward further augmenting the cytotoxic potential of amiloride by evaluating the potency of other C(5) derivatives.

Surprisingly, we observed that although UCD38B induces an autophagic response, its mechanism of cytotoxicity is via AIF-dependent programmed necrosis or type III PCD. While necrotic cell death has traditionally been conceptualized as a non-specific process, a series of recent studies has revealed that necrosis can be divided into different subtypes, some of which are regulated and may be exploited for therapeutic benefit [29]. Many of the morphological and biochemical characteristics of these subtypes are reflected in UCD38B-treated breast cancer cells, however, none of the previously described programmed necrosis mechanisms account for all of the changes that we observe in breast tumor cells after UCD38B treatment.

Parthanatos and necroptosis are recently described forms of type III PCD that are characterized by AIF translocation, but they differ in the mechanisms underlying flavoprotein release from the mitochondria. In parthanatos PARP processing promotes the formation of PAR polymers, which translocate to the mitochondria from the nucleus to promote AIF release [30]. AIF release in necroptosis is triggered by various stimuli including calpains, cathepsins, reactive oxygen species, and Bax, and is dependent on the RIP1 kinase [29], [31]. However, our observations that 38B-induced cell death is not affected by PARP or calpain inhibitors (not shown), or the RIP1 inhibitor necrostatin-1 (Figures 8B and 8C), rule out these two mechanisms.

Paraptosis is a type III PCD that is characterized by extensive vacuolation and swelling of the endoplasmic reticulum and mitochondria, and caspase-independent cell death [8]. Methuosis, another type III PCD, is also characterized by the presence of large cytosolic vacuoles that are formed through macropinocytosis by clathrin-independent endocytic vesicle trafficking [32]. In contrast, amiloride has been demonstrated to inhibit macropinocytosis through its inhibition of NHE1 [33]. Like UCD38B, both paraptosis and methuosis can produce an autophagic response that is not required for cell death, however a role of AIF in these cell death processes has not been described [34], [35]. Thus, the precise mechanism of UCD38B-induced programmed necrosis remains to be delineated.

A key question that emerges from our studies concerns the molecular target of UCD38B action. The anti-cancer effects of amiloride have been ascribed to its inhibition of sodium-hydrogen exchanger (NHE1) and urokinase plasminogen activator (uPA) [12], however, our previous studies have ruled out NHE1. While uPA remains a possibility, our observations point to a strong correlation between cytotoxic potency of the amiloride derivatives and their cell permeability. Thus, either UCD38B acts on an intracellular uPA form that harbors protease activity and is essential for tumor cell viability, or an unknown intracellular target remains to be identified. Our current studies are directed toward resolving these possibilities.

In summary, our studies provide the impetus for the development of new amiloride-based anti-cancer small molecules that initiate cell cycle-independent type III cell death in cancer cells. As these agents act on cells independent of their proliferative state, we predict that they would act synergistically with radiation therapy and conventional chemotherapies that target proliferating cancer cells. Moreover, slowly proliferating or dormant cancer cells could be effectively targeted by this class of compounds. Finally, cancer cells that are resistant to apoptosis-inducing drugs, including tumor-initiating or cancer stem cells, may also be targeted by this class of necrosis-inducing agents.

## References

[1] Aguirre-Ghiso JA (2007) Models, mechanisms and clinical evidence for cancer dormancy. Nat Rev Cancer 7: 834–846. 17957189 10.1038/nrc2256 PMC2519109

[2] Wilson TR, Longley DB, Johnston PG (2006) Chemoresistance in solid tumours. Ann Oncol 17 Suppl 10x315–324. 17018746 10.1093/annonc/mdl280

[3] Ricci MS, Zong WX (2006) Chemotherapeutic approaches for targeting cell death pathways. Oncologist 11: 342–357. 16614230 10.1634/theoncologist.11-4-342 PMC3132471

[4] Schweichel JU, Merker HJ (1973) The morphology of various types of cell death in prenatal tissues. Teratology 7: 253–266. 4807128 10.1002/tera.1420070306

[5] Clarke PG (1990) Developmental cell death: morphological diversity and multiple mechanisms. Anat Embryol (Berl) 181: 195–213. 2186664 10.1007/BF00174615

[6] Yuan J, Lipinski M, Degterev A (2003) Diversity in the mechanisms of neuronal cell death. Neuron 40: 401–413. 14556717 10.1016/s0896-6273(03)00601-9

[7] Gozuacik D, Kimchi A (2007) Autophagy and cell death. Curr Top Dev Biol 78: 217–245. 17338918 10.1016/S0070-2153(06)78006-1

[8] Broker LE, Kruyt FA, Giaccone G (2005) Cell death independent of caspases: a review. Clin Cancer Res 11: 3155–3162. 15867207 10.1158/1078-0432.CCR-04-2223

[9] Codogno P, Meijer AJ (2005) Autophagy and signaling: their role in cell survival and cell death. Cell Death Differ 12 Suppl 21509–1518. 16247498 10.1038/sj.cdd.4401751

[10] Lorenzo HK, Susin SA (2007) Therapeutic potential of AIF-mediated caspase-independent programmed cell death. Drug Resist Updat 10: 235–255. 18180198 10.1016/j.drup.2007.11.001

[11] Moubarak RS, Yuste VJ, Artus C, Bouharrour A, Greer PA, et al (2007) Sequential activation of poly(ADP-ribose) polymerase 1, calpains, and Bax is essential in apoptosis-inducing factor-mediated programmed necrosis. Mol Cell Biol 27: 4844–4862. 17470554 10.1128/MCB.02141-06 PMC1951482

[12] Matthews H, Ranson M, Kelso MJ (2011) Anti-tumour/metastasis effects of the potassium-sparing diuretic amiloride: An orally active anti-cancer drug waiting for its call-of-duty? Int J Cancer 129: 2051–2061. 21544803 10.1002/ijc.26156

[13] Hegde M, Roscoe J, Cala P, Gorin F (2004) Amiloride kills malignant glioma cells independent of its inhibition of the sodium-hydrogen exchanger. J Pharmacol Exp Ther 310: 67–74. 15010500 10.1124/jpet.103.065029

[14] Harley W, Floyd C, Dunn T, Zhang XD, Chen TY, et al (2010) Dual inhibition of sodium-mediated proton and calcium efflux triggers non-apoptotic cell death in malignant gliomas. Brain Res 1363: 159–169. 20869350 10.1016/j.brainres.2010.09.059 PMC2996276

[15] Garcia-Canero R, Trilla C, Perez de Diego J, Diaz-Gil JJ, Cobo JM (1999) Na+ :H+ exchange inhibition induces intracellular acidosis and differentially impairs cell growth and viability of human and rat hepatocarcinoma cells. Toxicol Lett 106: 215–228. 10403666 10.1016/s0378-4274(99)00072-7

[16] Yang X, Wang D, Dong W, Song Z, Dou K (2010) Inhibition of Na(+)/H(+) exchanger 1 by 5-(N-ethyl-N-isopropyl) amiloride reduces hypoxia-induced hepatocellular carcinoma invasion and motility. Cancer Lett 295: 198–204. 20338684 10.1016/j.canlet.2010.03.001

[17] Yang X, Wang D, Dong W, Song Z, Dou K (2011) Expression and modulation of Na(+)/H(+) exchanger 1 gene in hepatocellular carcinoma: A potential therapeutic target. J Gastroenterol Hepatol 26: 364–370. 21261728 10.1111/j.1440-1746.2010.06382.x

[18] Rich IN, Worthington-White D, Garden OA, Musk P (2000) Apoptosis of leukemic cells accompanies reduction in intracellular pH after targeted inhibition of the Na(+)/H(+) exchanger. Blood 95: 1427–1434. 10666221

[19] Massey AP, Harley WR, Pasupuleti N, Gorin FA, Nantz MH (2012) 2-Amidino analogs of glycine-amiloride conjugates: inhibitors of urokinase-type plasminogen activator. Bioorg Med Chem Lett 22: 2635–2639. 22366654 10.1016/j.bmcl.2011.12.123 PMC3329872

[20] Palandoken H, By K, Hegde M, Harley WR, Gorin FA, et al (2005) Amiloride peptide conjugates: prodrugs for sodium-proton exchange inhibition. J Pharmacol Exp Ther 312: 961–967. 15509720 10.1124/jpet.104.076984

[21] Mandlekar S, Hebbar V, Christov K, Kong AN (2000) Pharmacodynamics of tamoxifen and its 4-hydroxy and N-desmethyl metabolites: activation of caspases and induction of apoptosis in rat mammary tumors and in human breast cancer cell lines. Cancer Res 60: 6601–6606. 11118041

[22] Bursch W, Ellinger A, Gerner C, Frohwein U, Schulte-Hermann R (2000) Programmed cell death (PCD). Apoptosis, autophagic PCD, or others? Ann N Y Acad Sci 926: 1–12. 10.1111/j.1749-6632.2000.tb05594.x 11193023

[23] Meley D, Bauvy C, Houben-Weerts JH, Dubbelhuis PF, Helmond MT, et al (2006) AMP-activated protein kinase and the regulation of autophagic proteolysis. J Biol Chem 281: 34870–34879. 16990266 10.1074/jbc.M605488200

[24] Shintani T, Klionsky DJ (2004) Autophagy in health and disease: a double-edged sword. Science 306: 990–995. 15528435 10.1126/science.1099993 PMC1705980

[25] Deng JY, Chen SJ, Jow GM, Hsueh CW, Jeng CJ (2009) Dehydroeburicoic acid induces calcium- and calpain-dependent necrosis in human U87MG glioblastomas. Chem Res Toxicol 22: 1817–1826. 19848398 10.1021/tx9002275

[26] Xu X, Chua CC, Kong J, Kostrzewa RM, Kumaraguru U, et al (2007) Necrostatin-1 protects against glutamate-induced glutathione depletion and caspase-independent cell death in HT-22 cells. J Neurochem 103: 2004–2014. 17760869 10.1111/j.1471-4159.2007.04884.x

[27] Han W, Li L, Qiu S, Lu Q, Pan Q, et al (2007) Shikonin circumvents cancer drug resistance by induction of a necroptotic death. Mol Cancer Ther 6: 1641–1649. 17513612 10.1158/1535-7163.MCT-06-0511

[28] Joza N, Pospisilik JA, Hangen E, Hanada T, Modjtahedi N, et al (2009) AIF: not just an apoptosis-inducing factor. Ann N Y Acad Sci 15: 2–11. 10.1111/j.1749-6632.2009.04681.x 19723031

[29] Christofferson DE, Yuan J (2010) Necroptosis as an alternative form of programmed cell death. Curr Opin Cell Biol 22: 263–268. 20045303 10.1016/j.ceb.2009.12.003 PMC2854308

[30] Wang Y, Kim NS, Haince JF, Kang HC, David KK, et al.. (2011) Poly(ADP-ribose) (PAR) binding to apoptosis-inducing factor is critical for PAR polymerase-1-dependent cell death (parthanatos). Sci Signal 4. 10.1126/scisignal.2000902 PMC3086524 21467298

[31] Delavallee L, Cabon L, Galan-Malo P, Lorenzo HK, Susin SA (2011) AIF-mediated caspase-independent necroptosis: a new chance for targeted therapeutics. IUBMB Life 63: 221–232. 21438113 10.1002/iub.432

[32] Overmeyer JH, Young AM, Bhanot H, Maltese WA (2011) A chalcone-related small molecule that induces methuosis, a novel form of non-apoptotic cell death, in glioblastoma cells. Mol Cancer 10: 69. 21639944 10.1186/1476-4598-10-69 PMC3118192

[33] Koivusalo M, Welch C, Hayashi H, Scott CC, Kim M, et al (2010) Amiloride inhibits macropinocytosis by lowering submembranous pH and preventing Rac1 and Cdc42 signaling. J Cell Biol 188: 547–563. 20156964 10.1083/jcb.200908086 PMC2828922

[34] Overmeyer JH, Kaul A, Johnson EE, Maltese WA (2008) Active ras triggers death in glioblastoma cells through hyperstimulation of macropinocytosis. Mol Cancer Res 6: 965–977. 18567800 10.1158/1541-7786.MCR-07-2036 PMC2994605

[35] Samadder P, Bittman R, Byun HS, Arthur G (2009) A glycosylated antitumor ether lipid kills cells via paraptosis-like cell death. Biochem Cell Biol 87: 401–414. 19370058 10.1139/o08-147

